# Supplementary material for: Continuous control of classical-quantum crossover by external high pressure in the coupled chain compound CsCuCl3
Source: Nat Commun. 2021 Jul 12;12:4263. doi: 10.1038/s41467-021-24542-6 (PMC8275658; doi:10.1038/s41467-021-24542-6)
Supplement: Supplementary file 1 — Supplementary information [file 41467_2021_24542_MOESM1_ESM.pdf]

Supplementary Information: "Continuous control of classical-quantum crossover by external high pressure in the coupled chain compound $CsCuCl_3$"

Daisuke Yamamoto[1,2], Takahiro Sakurai[3], Ryosuke Okuto[4], Susumu Okubo[5], Hitoshi Ohta[5], Hidekazu Tanaka[6], and Yoshiya Uwatoko[7]
[1]*Department of Physics, Nihon University, Tokyo 156-8550, Japan*
[2]*Department of Physics and Mathematics, Aoyama Gakuin University, Kanagawa 252-5258, Japan*
[3]*Research Facility Center for Science and Technology, Kobe University, Kobe 657-8501, Japan*
[4]*Graduate School of Science, Kobe University, Kobe 657-8501, Japan*
[5]*Molecular Photoscience Research Center, Kobe University, Kobe 657-8501, Japan*
[6]*Department of Physics, Tokyo Institute of Technology, Meguro-ku, Tokyo 152-8551, Japan and*
[7]*Institute for Solid State Physics, The University of Tokyo, Chiba 277-8581, Japan*

Supplementary Note 1: Transformation into a twisted spin frame

In the laboratory frame, the coupled-chain triangular-lattice antiferromagnet (TLAF) $CsCuCl_3$ in a magnetic field H parallel to the c axis is well described by the following Hamiltonian [1]:

$$\begin{aligned}\hat{\mathcal{H}} &= -2J_0^{\perp}\sum_{i,n}\left(\hat{s}_{i,n}^{x}\hat{s}_{i,n+1}^{x}+\hat{s}_{i,n}^{y}\hat{s}_{i,n+1}^{y}\right)-2J_0^{\parallel}\sum_{i,n}\hat{s}_{i,n}^{z}\hat{s}_{i,n+1}^{z}-\boldsymbol{D}\cdot\sum_{i,n}\left(\hat{\boldsymbol{s}}_{i,n}\times\hat{\boldsymbol{s}}_{i,n+1}\right)\\ &\quad +2J_1\sum_{\langle i,j\rangle,n}\hat{\boldsymbol{s}}_{i,n}\cdot\hat{\boldsymbol{s}}_{j,n}-H\sum_{i,n}\hat{s}_{i,n}^{z},\end{aligned}\qquad(1)$$

where $\hat{\boldsymbol{s}}_{i,n}$ denotes the local $S=1/2$ spin on site i of the n-th triangular layer. The ferromagnetic interaction between the transverse (longitudinal) components of the nearest-neighbour spins along the c axis is denoted by $J_0^{\perp}$ ($J_0^{\parallel}$) and the isotropic antiferromagnetic interaction between the spins in the ab plane is J_1. The Dzyaloshinskii-Moriya (DM) interaction $\boldsymbol{D}=(0,0,d)$, which favors helical spin structures along the c axis, can be eliminated by the unitary transformation [2]

$$\begin{pmatrix}\hat{s}_{i,n}^{x}\\ \hat{s}_{i,n}^{y}\\ \hat{s}_{i,n}^{z}\end{pmatrix}=\begin{pmatrix}\cos nq & -\sin nq & 0\\ \sin nq & \cos nq & 0\\ 0 & 0 & 1\end{pmatrix}\begin{pmatrix}\hat{S}_{i,n}^{x}\\ \hat{S}_{i,n}^{y}\\ \hat{S}_{i,n}^{z}\end{pmatrix}\qquad(2)$$

with the twist angle q along the c axis. By setting $q=\arctan(d/2J_0^{\perp})$, we can rewrite the Hamiltonian in the form with no DM term as

$$\hat{\mathcal{H}} = -2J_0\sum_{i,n}\left(\hat{\boldsymbol{S}}_{i,n}\cdot\hat{\boldsymbol{S}}_{i,n+1}-\Delta_0\hat{S}_{i,n}^{z}\hat{S}_{i,n+1}^{z}\right)+2J_1\sum_{\langle i,j\rangle,n}\hat{\boldsymbol{S}}_{i,n}\cdot\hat{\boldsymbol{S}}_{j,n}-H\sum_{i,n}\hat{S}_{i,n}^{z},$$

which is Eq. (1) of the main text. The parameters are related to those of the original Hamiltonian by $J_0=J_0^{\perp}\sqrt{1+(d/2J_0^{\perp})^2}$ and $J_0(1-\Delta_0)=J_0^{\parallel}$. In the main text, we determine the pressure dependencies of the three parameters J_0, Δ_0, and J_1 of the model Hamiltonian in the twisted spin space. Note that if the magnetic field has a finite transverse component (say, $\propto\sum_{i,n}\hat{s}_{i,n}^{x}$), this unitary transformation cannot simplify the model since one has to deal with a non-uniform magnetic field with different directions for different layers ($\propto\sum_{i,n}(\hat{S}_{i,n}^{x}\cos nq-\hat{S}_{i,n}^{y}\sin nq)$) as a trade-off for eliminating the DM term.

Supplementary Note 2: Differences in the pressure environment from the previous experiments by Sera et al.

In the comparison of the theoretical magnetization curves with the experimental data shown in Fig. 7, the agreement seems to get slightly worse for larger values of pressure, especially at $P=0.9$ GPa (Fig. 7d). A possible reason for this is a slight underestimation of pressure due to the pressure inhomogeneity in the sample used in Ref. [3].

The large differences in the experimental conditions from the previous experiment by Sera et al. are the amount of sample and the setting way of tin whose superconducting transition temperature is used for pressure calibration at low temperature [4]. As for the amount of sample, we used a sample of 17.7 mg, while Sera et al. used a sample of

62.5 mg to gain the S/N ratio in their homemade magnetization measurement equipment [3]. Since the dimensions of the pressure cells are similar, this causes a difference in sample length of about 3 times. In general, the pressure inhomogeneity in a sample becomes larger as the sample gets longer since it occurs along the cylindrical axis in the piston-cylinder type pressure cell when pressure-transmitting fluid freezes. Therefore, it can be said that the pressure inhomogeneity in the sample was expected to be larger in the previous experiment by Sera et al. [3], compared to that in the measurement of the present work. Regarding the setting of tin, we made a tube with almost the same height as the sample, and put the sample into the tin tube (see Methods in the main text) in order to correctly detect the pressure experienced by the sample. On the other hand, Sera et al. placed tin just below the sample [M. Sera, private communication]. It means that they measured the pressure only at the bottom of the sample with larger pressure distribution.

Supplementary Fig. 2 shows the comparison of the pressure dependence of the reciprocal of slope of low-field magnetization curves. It can be seen that the value obtained from the experiment by Sera et al. [3] becomes clearly larger than that from our experiment for high pressures ($\gtrsim 0.75$ GPa). Indeed, this fact suggests that the pressure value measured in the experiment by Sera et al. may be slightly underestimated in the high pressure region, owing to the pressure inhomogeneity in their long sample and the above-mentioned measurement method of the pressure. Moreover, the pressure inhomogeneity could also make unclear the plateau structure and the inflection points in the experimental magnetization curve for high pressures.

---

[1] Tanaka, H., Schotte, U., & Schotte, K. ESR Modes in $CsCuCl_3$. *J. Phys. Soc. Jpn.* **61**, 1344 (1992).

[2] Nikuni T. & Shiba, H. Quantum Fluctuations and Magnetic Structures of $CsCuCl_3$ in High Magnetic Field. *J. Phys. Soc. Jpn.* **62**, 3268 (1993).

[3] Sera, A., Kousaka, Y., Akimitsu, J., Sera, M. & Inoue, K. Pressure-induced quantum phase transitions in the $S = 1/2$ triangular lattice antiferromagnet $CsCuCl_3$. *Phys. Rev. B* **96**, 014419 (2017).

[4] Smith, T. F. & Chu, C. W. Will Pressure Destroy Superconductivity? *Phys. Rev.* **159**, 353 (1967).

[5] Lohmann, A., Schmidt, H.-J. & Richter J. Tenth-order high-temperature expansion for the susceptibility and the specific heat of spin-s Heisenberg models with arbitrary exchange patterns: Application to pyrochlore and kagome magnets. *Phys. Rev. B* **89**, 014415 (2014).

[6] Miyake, A., Shibuya, J., Akaki, M., Tanaka, H. & Tokunaga, M. Magnetic field induced polar phase in the chiral magnet $CsCuCl_3$. *Phys. Rev. B* **92**, 100406(R) (2015).

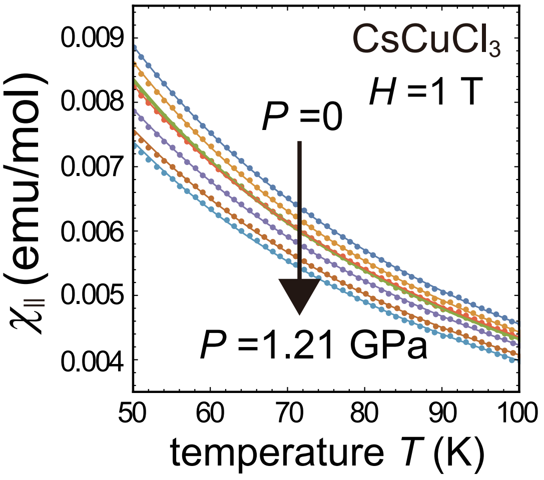

Supplementary Fig 1: **Fitting for magnetic susceptibility under pressure.** Enlarged view of Fig. 4a in the temperature range $50 < T < 100$ K together with the best-fitting theoretical curves (solid lines) according to the tenth-order high-temperature expansion [5] combined with the Padé approximation.

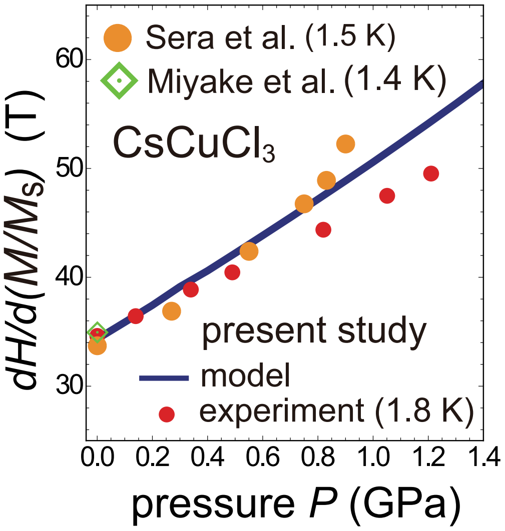

Supplementary Fig 2: **Fitting for magnetization curves under pressure.** The reciprocal of the slopes of the low-field magnetization curves shown in Fig. 4b is plotted as a function of pressure P, together with the ones extracted from the experiments of Miyake et al. [6] and Sera et al. [3]. The theoretical curve obtained with the model parameters of Eqs. (6-8) is plotted by the solid line.
